# Multiple Cultures and Extended Incubation for Upper Extremity Revision Arthroplasty Affect Clinical Care: A Cohort Study

**DOI:** 10.5435/JAAOSGlobal-D-19-00150

**Published:** 2019-11-13

**Authors:** Jared Mahylis, Alexander DeHaan, Zachary B. Domont, Austin R. Thompson, Robert M. Orfaly, Penelope Barnes, Adam J. Mirarchi

**Affiliations:** From the Specialty Physicians of Illinois, Olympia Fields, IL (Dr. Mahylis); the Orthopaedic + Fracture Specialists, Portland, OR (Dr. DeHaan); the Lincolnshire Orthopedics, Lincolnshire, IL (Dr. Domont); the Department of Orthopaedics and Rehabilitation, Oregon Health & Science University, Portland, OR (Mr.Thompson, Dr. Orfaly, Dr. Mirarchi); and the Infectious Disease, PeaceHealth Medical Group—Whatcom, Bellingham, WA (Dr. Barnes).

## Abstract

Skin flora organisms (SFOs) isolated from 1 to 2 tissue samples during shoulder and elbow revision arthroplasty are difficult to distinguish as contamination or infection. We examined the change in clinical care after implementation of an Arthroplasty Infection Protocol by increasing the number of intraoperative samples held for 10-day incubation to a minimum of 5.

**Methods:**

Infection was defined as ≥3 cultures growing the same SFO or any one culture growing any other virulent organism. SFOs growing in 1 to 2 samples were defined as skin flora contaminant. All cases were compared with pre–Arthroplasty Infection Protocol institution standard to determine changes in microbiological diagnosis and resultant antibiotic treatment.

**Results:**

Forty cases fulfilled the inclusion criteria: 50% of these were culture negative, and 35% grew *Propionibacteria*. When compared with the standard of obtaining one sample, this protocol altered the microbiological diagnosis and subsequent antibiotic treatment in 45% of cases (95% confidence interval 29% to 62%). This protocol had a predictive value of joint sterility in 95% of culture-negative cases (95% confidence interval 74% to 99%).

**Discussion:**

The addition of 5 or more samples held for 10-day incubation reliably differentiated between joint infection, contamination, and sterility, which changed the course of care in 45% of surgical cases.

Incidence of infection is less than 2% in primary shoulder arthroplasty cases^1,2,3,4,5^ and 3% to 7.5% in primary total elbow arthroplasty cases.^6,7,8,9,10,11,12,13^ Rates increase with upper extremity arthroplasty revision.^2,5^ Upper extremity prosthetic joint infection (PJI) caused by the indolent skin flora organisms (SFOs), namely coagulase-negative *Staphylococcus* (CoNS), *Corynebacteria* species, and *Propionibacteria*, is particularly challenging to diagnose. These organisms elicit minimal signs of local or systemic infection.^14,15,16,17^ A single positive intraoperative culture is of low diagnostic value in identifying the organism as a true pathogen versus a culture contaminant.^18,19^ Sensitivity of cultures for *any* bacteria in a PJI is, at best, 65%.^18^

Studies of lower limb arthroplasty revision demonstrate that multiple tissue samples improve sensitivity, aiding in distinguishing between infection and contamination.^18,19,20,21^ Short-term culture incubation misses 25% to 50% of infections, particularly those caused by organisms of low virulence.^16,22,23^ Current literature recommends obtaining a minimum of three and ideally 5 to 6 tissue samples and maintaining cultures for 5 to 14 days.^24–26^ In cases with negative culture growth but consistent with a clinical picture of PJI, the cultures may be maintained for longer than 14 days.^25^ The Musculoskeletal Infection Society (MSIS) criteria for the diagnosis of hip and knee PJI were last revised in 2018.^26^ The effect of a multiple tissue culture protocol for upper extremity arthroplasty revision on patient care remains unknown. Our institution developed an Arthroplasty Infection Protocol (AIP) for the diagnosis of PJIs. Analysis of hip and knee revision arthroplasty showed that the protocol changed microbiological diagnosis and antibiotic treatment in 34% and 30% of cases, respectively.^20^ The current analysis was a pilot study to determine the effect of the AIP, previously assessed in an infectious setting of the lower extremity, now applied to cases of upper extremity revision arthroplasty. Thus, we asked whether 5 or more cultures incubated for 10 days would change microbiological diagnosis and antibiotic treatment.

## Methods

### Arthroplasty Infection Protocol

The AIP was established in 2010 as a quality improvement project by the orthopaedics and infectious diseases (IDs) departments in our university-based tertiary referral center. A surgical tray with six separate sets of rongeurs and forceps is used to obtain a minimum of five tissue samples from sites adjacent to or underneath the implant during revision arthroplasty. Specific sample location is determined by the primary surgeon. Samples are taken with a separate rongeur and handled solely by the surgeon to avoid microbial cross-contamination. No culture swabs are allowed. Individual samples are placed into separate sterile collection bottles using sterile forceps and are sent to the laboratory for analysis. Samples are separately cultured into aerobic media and into thioglycolate enrichment broth for 10 days. Aspirated joint fluid, considered to be a separate tissue sample, is directly inoculated into blood culture bottles and subsequently incubated for 5 days. Intraoperative frozen section of one sample provides an analysis of neutrophil cell count per high-powered field, with the final histopathological diagnosis also available when completed. Standard perioperative antibiotic prophylaxis (cefazolin or appropriate alternative) is administered after samples are taken.

SFOs are defined as CoNS, *Corynebacteria*, and *Propionibacteria*. Cultured SFOs are defined as contaminant if there is a positive culture of the same SFO in 1 to 2 of the 5 cultures, in which case the joint is considered “not clinically infected” and the patient is not placed on a prolonged antibiotic regimen. Growth of 3+ of the 5 cultures with the same SFO is defined as a PJI,^18^ and preliminary antibiotic treatment is initiated. Growth in 1+ culture of any non-SFO is defined as a virulent organism (VO), and the patient is subsequently treated for a PJI. When none of the five cultures grow bacteria and the patient is not treated, the joint is considered sterile. Tissue samples are not routinely cultured for acid-fast bacteria or fungal organisms, unless the surgeon has increased clinical suspicion.^27^ Interpretation of the results and management of patients' postoperative course are conducted in a multidisciplinary orthopaedic-infectious disease clinic.

### Retrospective Arthroplasty Infection Protocol Data Review

This observational cohort study was conducted with institutional review board approval. Study inclusion criteria were all of the following:

(1) shoulder or elbow prosthetic joint revision between February 1, 2010, and April 30, 2012;
(2) five or more tissue cultures held for 10-day incubation;
(3) at least 1 year of clinical follow-up; and
(4) no antibiotics taken in the month before revision surgery (excluding a single dose of perioperative antibiotics).

Chart review of the ID database identified 114 patients who underwent 124 cases of shoulder (n = 105) and elbow (n = 19) revision arthroplasty during the study period. Eighty-three cases (80 patients) were excluded because they had <5 tissue samples taken or cultures were not held for 10 days; one patient was excluded because they received antibiotics within the month before surgery. Thus, 40 cases in 33 patients were reviewed (Table 1). Shoulder procedures accounted for 77.5% (n = 31) of cases, and 22.5% (n = 9) involved the elbow. Fifty percent of cases had one or more previous revisions before this index surgery. A median of 24 months (range 0.5 to 110) had elapsed from the time of their primary or previous revision arthroplasties until the arthroplasty revision at our institution. No patients died within 1 year of revision surgery.

**Table 1:** Patient Characteristics

Patients	33
Cases	40
Female (n, %)	15 (48%)
Age (yr)	
Median (range)	66 (30-80)
Body mass index	
Median (range)	31 (20-54)
Type of arthroplasty (cases)	
TSA (n, %)	17 (42.5%)
Shoulder hemi (n, %)	14 (35%)
TEA (n, %)	9 (22.5%)
No. of previous revisions (n, %)	
0	20 (50%)
1	12 (30%)
≥2	8 (20%)
Time since last surgery (mo)	
Median (range)	24 (0.5-110)
Microbiology (n)	
*Propionibacteria* only	8
CoNS only	3
*Enterococcus* only	2
MSSA only	1
Polymicrobial^a^	6
Culture negative	20

CoNS = coagulase-negative *Staphylococcus*, hemi = hemiarthroplasty, MSSA = methicillin-sensitive *Staphylococcus aureus*, TEA = total elbow arthroplasty, TSA = total shoulder arthroplasty

a Polymicrobial represents two or more organisms grown in the same case.

All revision surgeries were performed at our institution, although the previous surgeries may have been done elsewhere. Some patients had more than one revision during the study period; each revision was treated independently for analysis. Medical records were examined for demographic information, surgical history, microbial and antibiotic history, pathology results, and clinical follow-up.

### Assumptions/Definitions

All cases were compared with the pre-AIP institutional standard of obtaining only one intraoperative tissue sample. Thus, two assumptions were made. First, obtaining a single tissue sample would have not allowed for differentiation between a contaminant and pathogen SFO. However, if multiple samples were taken, this distinction was more easily made. Second, if only one sample was taken, and yielded a VO, the patient would be subsequently treated for this pathogen as the cause of the PJI. If multiple samples were taken and all resulted in growth of the same VO, the patient would similarly be treated for this pathogen as the cause of the PJI. However, if ≤4 samples grew that same VO, there was the possibility that the organism would have been missed if only one sample had hypothetically been taken. Using these assumptions, culture results could be placed into five categories (labeled as A through E) as outlined in Table 2.

**Table 2:** AIP Categories of Monomicrobial Culture Growth: Effect on Microbiological Diagnosis and Antibiotic Treatment

Category A	Definite SFO contaminant
	1-2 of 5 positive cultures for the same SFO
	*Does this cause a change in microbiological Dx compared with only one sample?*: Yes
	*Does this change antibiotic treatment:* Yes
	Result: Patient not treated for infection that would have been treated with only one positive culture
Category B	Definite SFO pathogen
	3-5 of 5 positive cultures for the same SFO
	*Does this cause a change in microbiological Dx compared with only one sample?*: Yes
	*Does this change antibiotic treatment:* Yes
	Result: Patient treated for infection that would have not been treated with only one negative culture
Category C	VO infection in 100% of cultures
	VO grown in all 5 cultures taken
	*Does this produce a change in microbiological Dx?*: No
	*Does this change antibiotic treatment?:* No
	Result: Patient treated for infection as per one positive culture
Category D	Potentially “missed” VO infection
	VO grown but less than 5 out of 5 cultures show VO growth
	*Does this cause a change in microbiological Dx compared with only one sample?:* Yes
	*Does this change antibiotic treatment?:* Yes
	Result: Patient treated for infection that would not have been treated with only one negative culture
Category E	Sterile joints
	0 of 5 cultures with any growth
	*Does this produces a change in microbiological Dx?*: No
	*Does this change antibiotic treatment?:* No
	Result: Patient not treated for infection as per one negative culture

AIP = Arthroplasty Infection Protocol, SFO = skin flora organism, included coagulase-negative *Staphylococcus* (CoNS), *Corynebacteria* species, and *Propionibacteria*, VO = virulent organism, included any cultured organism not a skin flora organism

The AIP compared with the standard of only taking one intraoperative culture: (1) Change in microbiological diagnosis defined as when the AIP would allow for the determination of an organism as a true pathogen causing the PJI or a culture contaminant that could not be distinguished with only one intraoperative culture. (2) Change in antibiotic treatment defined as when the AIP would allow for the ability to distinguish cultured bacteria as either pathogen or contaminant and the subsequent ability to narrow or withhold appropriate antibiotics.

### Effect on Clinical Care

The AIP's effect on clinical care was measured by assessing the change in microbiological diagnosis and antibiotic use when compared with the pre-AIP institution standard.^20^ Based on categories A to E, the AIP would change the microbiological diagnosis in those organisms defined as SFO contaminants (A), SFO pathogens (B), or potentially “missed” VOs (D). Notably, the AIP would not change the microbiological diagnosis in cases with 100% VO culture growth (C) or those with no growth and deemed a “sterile joint” (E).

The effect of the AIP on a change in antibiotic usage was assessed using the same criteria. If antibiotics were withheld because the cultured SFO was defined as a contaminant rather than a pathogen (A), this was considered a change in antibiotic management. If antibiotics were prescribed for an organism deemed an SFO pathogen (B) or a potentially “missed” VO (D), this was also classified as a change in antibiotic treatment. However, for cases with 100% VO culture growth (C) or “sterile joints” (E), the AIP did not alter antibiotic management as opposed to a situation in which only one sample was taken.

For any individual, potential for polymicrobial culture growth exists (multiple SFOs, multiple VOs, or a combination of VOs and SFOs). In these circumstances, the previous definitions were applied to each organism but were only counted as a change in the microbiological diagnosis once per case to avoid falsely elevating the numbers. Similarly, antibiotic treatment took into account the additional organisms cultured, and antibiotics were prescribed against both organisms. For instance, antibiotics directed at an SFO pathogen or VO could provide unintended coverage against a polymicrobial SFO contaminant. In this circumstance, the AIP would have changed the antibiotic treatment for the SFO pathogen or VO but not the SFO contaminant, as there was unintended coverage regardless of the true microbiological diagnosis.

The AIP's effect on changing the microbiological diagnosis was calculated by adding definite SFO contaminants (A), SFO pathogens (B), and cases of potentially “missed” VOs (D). The result was expressed as a percent of the total number of cases analyzed (A through E), with 95% confidence intervals (CIs) calculated by exact methods. This same formula was used to determine the overall effect on antibiotic administration for cases of SFO contaminants that avoided antibiotics (A), SFO pathogens with narrowed antibiotics (B), and all cases of potentially “missed” VOs (D).

### Follow-up

Orthopaedic follow-up continued for a minimum of 1 year from index revision surgery. Patients had routine orthopaedic and ID visits at 2 and 6 weeks postoperatively. Some patients were followed up solely by the orthopaedic department beyond 6 weeks if medically “cleared” by the ID team.

## Results

### Microbiology

Twenty of 40 cases were culture negative, and 20 of 40 cases were culture positive (Table 1). Fourteen culture-positive cases were monomicrobial, and 6 were polymicrobial. Fourteen of 20 culture-positive cases (35%; 11 shoulder and 3 elbow) grew *Propionibacteria*; of which, 8 were only *Propionibacteria* and 6 were *Propionibacteria* and at least one other organism.

### Effect on Microbiological Diagnosis

Six of the 20 cases with positive cultures were determined to be monomicrobial SFO contaminants (Table 3; cases 12 to 17), whereas 5 were deemed monomicrobial SFO pathogens (cases 1, 4, 5, 7, and 8). One case was identified as a monomicrobial potentially “missed” VO (case 20). Six cases grew polymicrobial flora (cases 2, 3, 6, 9, 10, and 11). We distinguished polymicrobial SFOs as either pathogen or contaminant (cases 3, 9, and 10) and cases with both SFOs and potentially “missed” VOs (cases 2, 6, and 11).

**Table 3:** Cultured Organisms: Culture and Pathology Results, Management, and 1-Year Outcome in 20 Cases With Positive Cultures

Patient Number	Case Number	Arthroplasty Type	No. of Previous Revisions	Total Cultures Taken	SFO Definite Pathogen (# + Cultures)	Virulent Organism (# + Cultures)	SFO Definite Contaminant (# + Cultures)	Pathology	Frozen (#PMNs/hpf)	CRP	ESR	CC	MSIS Definition	Antibiotics			Outcome
														Specific to SFO	Avoided	Specific to Virulent Organism	
1	1	TSA	≥2	5	Propi (3/5)			No acute inf	<1/hpf	0.5	29	NA	Inf	Yes			Doing well with abx spacer in place
1	2	TSA	≥2	5		MRSA (3/5)	Propi (2/5)	NA	NA	0.5	29	NA	Inf			Yes**	Doing well with abx spacer in place
2	3	TSA	1	6	Propi (6/6)		CoNS (1/6)	No acute inf	<1/hpf	0.5	25	NA	Inf	Yes			New TSA, on lifelong po abx
2	4	TSA	1	5	Propi (3/5)			No acute inf	NA	0.5	10	NA	Inf	Yes			New TSA, on lifelong po abx
3	5	TSA	0	6	Propi (6/6)			No acute inf	NA	2	40	NA	Inf	Yes			New S. Hemi, on lifelong po abx
3	6	TSA	1	6	Propi (4/6)	Actinomyces (1/6)	CoNS (1/6)	No acute inf	NA	2	40	NA	Inf			Yes**	New S. Hemi, on lifelong po abx
4	7	S. Hemi	≥2	6	Propi (4/6)			No acute inf	<1/hpf	NA	NA	NA	Inf	Yes			New TSA, on lifelong po abx
5	8	S. Hemi	1	6	Propi (4/6)			No acute inf	<1/hpf	NA	NA	NA	Inf	Yes			New S. Hemi, on lifelong po abx
6	9	TEA	1	8	Propi (8/8)		CoNS (1/8)	Acute inf	>10/hpf	5.1	95	NA	Inf	Yes			New TEA, off abx
7	10	TSA	0	7	CoNS (4/7)		Propi (1/7)	NA	NA	0.5	25	NA	Inf	Yes			New TSA, off abx
8	11	S. Hemi	0	7	Propi (6/7)	*Enterococcus* (4/7)		Chronic inf	Up to 15/hpf	NA	NA	NA	Inf			Yes**	Doing well with abx spacer in place
9	12	S. Hemi	0	6			Propi (2/6)	NA	NA	NA	NA	NA	Inf*		Yes		New S. Hemi, off abx
10	13	TEA	1	6			Propi (1/6)	NA	<1/hpf	0.5	26	NA	NA		Yes		New TEA, off abx
11	14	TEA	0	6			CoNS (1/6)	No acute inf	<1/hpf	NA	NA	NA	NA		Yes		New infection 2 months postop (6/6 MSSA) requiring I&D, 1 stage exchange arthroplasty, lifelong po abx
12	15	TEA	1	6			Propi (1/6)	Chronic inf	1/hpf	NA	NA	NA	NA		Yes		New TEA, off abx
13	16	TEA	1	5			CoNS (2/6)	NA	NA	NA	NA	NA	Inf*		Yes		New TEA, off abx
14	17	S. Hemi	0	6			CoNS (2/6)	No acute inf	<1/hpf	NA	62	133	Inf*		Yes		New TEA, off abx
15	18	TEA	0	6		MSSA (6/6)		NA	NA	NA	NA	NA	Inf			Yes	New TEA, on lifelong po abx
16	19	TSA	0	6		*Enterococcus* (6/6)		Chronic inf	NA	NA	NA	NA	Inf			Yes	New TSA, off abx
17	20	TSA	0	6		*Enterococcus* (4/6)		Focal acute inf	NA	0.9	27	NA	Inf			Yes**	Doing well with abx spacer in place

#PMNs/hpf = number of polymorphonuclear neutrophils per high-powered field, CC = cell count, CoNS = coagulase-negative *Staphylococcus*, CRP = C-reactive protein, ESR = erythrocyte sedimentation rate, Inf = inflammation, MRSA = methicillin-resistant *Staphylococcus aureus*, MSIS = Musculoskeletal Infection Society, MSSA = methicillin-sensitive *Staphylococcus aureus*, NA = not available, po abx = oral antibiotics, Propi = *Propionibacteria*, S. Hemi = shoulder hemiarthroplasty, SFO = skin flora organism, TEA = total elbow arthroplasty, TSA = total shoulder arthroplasty,

Inf* = MSIS 2018 criteria define case as infected but the AIP does not, Yes** = virulent organisms treated with antibiotics with less than 100% culture growth.

Compared with taking a single sample, the AIP altered microbiological diagnosis in 18 of 40 cases (45.0%, 95% CI 29% to 62%) (6 cases of SFO contaminants only, 5 cases of SFO pathogens only, 1 case of potentially “missed” VOs, and 6 polymicrobial cases, Table 4).

**Table 4:** Change in Microbiological Diagnosis and Antibiotic Usage

	Category	No. of Cases	Case Numbers	Percentage of Cases	95% Confidence Interval
Protocol changed microbiological diagnosis	Monomicrobial				
	SFO contaminant	6	12, 13, 14, 15, 16, 17	15.0	
	SFO pathogen	5	1, 4, 5, 7, 8	12.5	
	Potentially “missed” VO	1	20	2.5	
	Polymicrobial				
	Multiple SFOs, defined as either pathogen or contaminant	3	3, 9, 10	7.5	
	SFO and potentially “missed” VO	3	2, 6, 11	7.5	
	Total	18		45.0	29%-62%
Protocol changed antibiotic usage	1) SFO contaminant	6	12, 13, 14, 15, 16, 17	15.0	
	2) SFO pathogen	8	1, 3, 4, 5, 7, 8, 9, 10	20.0	
	3) Potentially “missed” VO	4	2, 6, 11, 20	10.0	
	Total	18		45.0	29%-62%

SFO = skin flora organism, SFOs included coagulase-negative *Staphylococcus* (CoNS), *Corynebacteria* species, and *Propionibacteria*, VO = virulent organism, VO included any cultured organism not an SFO

### Effect on Antibiotic Use

Six of 11 cases identified as SFO contaminant avoided unnecessary antibiotic treatment (Table 3; cases 12 to 17), of which 5 had no evidence of infection 1 year postoperatively. The remaining case (case 14) was found to have an infection with a different organism 2 months postoperatively (original; 1 of 5 CoNS, subsequent; 6 of 6 methicillin-sensitive *Staphylococcus aureus*) and was considered a new infection with a different organism after revision arthroplasty rather than a protocol failure. The five cases with SFO contaminants also had cultures of another organism; of which, two were virulent pathogen culture contaminants (cases 2 and 6) and three were of an SFO pathogen (cases 3, 9, and 10). These pathogens all required treatment with targeted antibiotics, and unintended coverage against the SFO contaminant was provided by this approach, eliminating additional antibiotic coverage.

Eight of 10 cases identified as SFO pathogen were treated with organism-specific antibiotics (Table 3; cases 1, 3 to 5, and 7 to 10); these cases had no evidence of recurrent infection at 1-year follow-up. The other 2 cases (6 and 11) that grew definite SFO pathogens also had growth of VOs and were given antibiotics that provided coverage of both the VO and the SFO. The AIP did not influence the antibiotic treatment in these two cases. In addition, all four cases of potentially “missed” VOs (cases 2, 6, 11, and 20) received antibiotics directed at the VO as a result of the AIP.

In summary, the AIP altered antibiotic usage in 6 cases of SFO contaminants, 8 cases of SFO pathogens, and 4 cases of potentially “missed” virulent pathogens (Table 4), consequently altering antibiotic usage in 18 of 40 cases (45.0%, 95% CI 29% to 62%).

### Arthroplasty Infection Protocol 1-Year Outcomes on Patients with Positive Cultures

We examined patient outcomes at least 1 year from index surgery to ensure that patients not treated for infection due to an SFO contaminant did not relapse with a defined “SFO contaminant.” Six patients had monomicrobial SFO contaminants; 5 of these had no evidence of infection up to 3 years after surgery. The remaining case (Table 3; case 14) was infected with a different organism 2 months postoperatively (original: 1 of 5 CoNS and subsequent: 6 of 6 methicillin-sensitive *Staphylococcus aureus*). This was considered a new infection at the time of revision arthroplasty rather than a protocol failure.

### Prediction of Joint Sterility

Twenty cases in 17 patients had “sterile joints” at the time of revision arthroplasty (Table 5). One (case 39) was treated beyond the scope of the protocol due to the patient's complicated medical and surgical history and given 6 weeks of antibiotics despite having negative cultures. This patient was excluded from our analysis. One case developed a postoperative infection 2 weeks after surgery (case 40); cultures were positive for both *Propionibacterium* (1 of 5) and *Actinobacterium* species (1 of 5), requiring further surgery and lifelong antibiotic suppression. At 1 year postoperatively, the remaining 18 revision arthroplasty cases were off antibiotics. Thus, the AIP had a 95% prediction of joint sterility in culture-negative cases (18 of 19 cases; 95% CI 74% to 99%).

**Table 5:** Culture-Negative Cases: Prediction of Joint Sterility in 20 Cases With No Positive Cultures

Patient Number	Case Number	Arthroplasty Type	No. of Previous Revisions	Total Cultures Taken	Organisms Cultured	Pathology	Frozen (#PMNs/hpf)	ABX Treatment?	Correct Treatment	1-yr Outcome
4	21	TSA	1	5	None	No acute inf	<2/hpf	No	Yes	New TSA, off abx
18	22	S. Hemi	≥2	6	None	Chronic inf	NA	No	Yes	New TSA, off abx
18	23	S. Hemi	≥2	6	None	No acute inf	<1/hpf	No	Yes	New TSA, off abx
19	24	TEA	1	7	None	No acute inf	<1/hpf	No	Yes	New TEA, off abx
19	25	TEA	≥2	5	None	No acute inf	<1/hpf	No	Yes	New TEA, off abx
20	26	S. Hemi	0	7	None	No acute inf	<1/hpf	No	Yes	New TSA, off abx
20	27	S. Hemi	1	5	None	No acute inf	<1/hpf	No	Yes	New TSA, off abx
21	28	TSA	0	6	None	No acute inf	<1/phf	No	Yes	New TSA, off abx
22	29	S. Hemi	0	6	None	No acute inf	<5/hpf	No	Yes	New S. Hemi, off abx
23	30	TSA	0	6	None	No acute inf	<1/hpf	No	Yes	New TSA, off abx
24	31	TSA	0	6	None	No acute inf	NA	No	Yes	New TSA, off abx
25	32	TSA	1	6	None	No acute inf	<1/hpf	No	Yes	New TSA, off abx
26	33	TSA	0	6	None	No acute inf	<10/hpf	No	Yes	New TSA, off abx
27	34	S. Hemi	0	6	None	No acute inf	<1/hpf	No	Yes	New S. Hemi, off abx
28	35	TSA	0	5	None	No acute inf	1/hpf	No	Yes	New TEA, off abx
29	36	S. Hemi	0	6	None	No acute inf	NA	Yes × 10 d, until cultures final	Yes	New S. Hemi, off abx
30	37	S. Hemi	0	6	None	No acute inf	<2/hpf	No	Yes	New TSA, off abx
31	38	TSA	0	6	None	No acute inf	<1/hpf	No	Yes	New TSA, off abx
32	39	S. Hemi	≥2	5	None	NA	NA	Yes × 6 wk, history of infection and osteomyelitis, high surgeon intraoperative suspicion	Yes	Doing well with antibiotic spacer in place (did require I&Ds and revision antibiotic spacer, subsequent cultures MSSA)
33	40	TEA	≥2	5	None	No acute inf	3/hpf	No	No	Required repeat I&D, lifelong abx suppression

#PMNs/hpf = number of polymorphonuclear neutrophils per high-powered field, abx = antibiotics, I&D = irrigation and débridement, Inf = inflammation, MSSA = methicillin-sensitive *Staphylococcus aureus*, NA = not available, po abx = oral antibiotics, S. Hemi = shoulder hemiarthroplasty, TEA = total elbow arthroplasty, TSA = total shoulder arthroplasty

One patient (patient 32, case 39) was treated beyond the scope of the protocol because of unique clinical history and given IV antibiotics despite negative cultures. She had a history of previous shoulder infection and radiographs concerning for osteomyelitis before her index procedure. After surgery, she was continued on IV antibiotics despite negative cultures and later required multiple I&Ds and a revision of her antibiotic spacer as cultures grew MSSA. One patient was determined to be a protocol failure. This case (patient 33, case 40) had 0 of 5 positive cultures but developed an infection 2 weeks postoperatively (cultures 1 of 5 *Actinobacterium* species and 1 of 5 *Propionibacteria*) that required an I&D with retention of implant. The decision was made to continue lifelong oral antibiotic suppression.

## Discussion

Our main finding was that the AIP altered microbiological diagnosis and antibiotic treatment in 45% of cases. In addition, joint sterility was predicted correctly 95% of the time. This is notable because accurate identification of positive cultures enables appropriate treatment of infected patients while preventing unneeded antibiotic use in uninfected patients. Institutional incidences of PJI are a nationally reported healthcare quality measure, which warrants the development of a protocol that improves diagnostic accuracy in these complex arthroplasty cases.

The methods were modeled on previous studies of lower extremity arthroplasty revision and culture requirements for more fastidious bacteria.^18,19,23^ AIP implementation altered antibiotic use in 45% of our patients, which notably affected patient care, resulting in continued institutional utilization of this protocol. Despite the complexity of our infection classification, we believe this protocol, which has shown benefit in our previous study,^20^ was translatable to assessment of bacterial infections in upper extremity revision arthroplasty.

The protocol for the diagnostic tests and definition of PJI in the AIP differs slightly from the MSIS 2018 criteria for hip and knee PJI. The MSIS definition requires a minimum of two positive cultures of the same organism^26^; the AIP requires three positive cultures of the same SFO or one positive culture of a VO. The MSIS criteria do not differentiate between SFOs and VOs^26^; there is some evidence to support that growth of a VO in a single specimen may represent PJI.^24^ Separating SFOs and VOs in the AIP also allows for easier determination for correct antibiotic treatment. The AIP maintained cultures for 10 days rather than up to 14; however, there are data to support that prolonging cultivation past 10 days did not improve sensitivity and may increase contamination in low VOs.^28^ Maintaining cultures for extended periods also increases costs and ties up valuable resources. In addition, the AIP did not rely on the minor criteria recommended in the 2018 definition of hip and knee PJI from MSIS. In the implementation of the AIP, preoperative serologic tests were often inconsistently obtained and excluded from the diagnostic algorithm.

One limitation is only 32% of cases met the full inclusion criteria. The reasons for this were twofold. Originally, miscommunications between the surgical team, operating room staff, and pathology teams resulted in mislabeled samples, combined samples, or incompletely analyzed samples. Most excluded cases were in the first implemented year of the AIP. Fewer cases were subsequently excluded. The hurdles experienced while implementing and ensuring this protocol's accuracy are important for other practices to consider. The second reason for the low inclusion number was that surgeons may have perceived certain patients as unlikely to be infected and, therefore, did not take all five samples, thus biasing our results for patients more likely to have infection. Furthermore, because this study was conducted before the implementation of the MSIS criteria, preoperative serologic laboratory test results were not explicitly a part of the AIP and, therefore, were inconsistently obtained.

We chose to combine shoulder and elbow arthroplasties, as was done in our previous study of lower extremity PJIs.^20^ Although there are differences in regard to likely bacterial pathogens in each anatomic joint, we felt the protocol would serve equally well in both the elbow and shoulder regardless of infectious organism. We recognize the small number of elbow cases in the study limits subgroup analysis but felt it necessary to include them to ensure consistency in AIP use.

Another limitation was the minimum 1-year follow-up time, as shoulder bacterial PJI may be delayed. Patients designated as infection free may have presented with signs or symptoms of infection beyond 1 year. However, owing to the large effect on antibiotic management and subsequent patient care, the authors thought it helpful to publish these short-term data. In addition, there is not a “benchmark” for diagnosing PJI,^26^ making it difficult to report sensitivity and specificity in our small cohort. Future research should aim to externally validate an algorithm using similar methods utilized in developing the 2018 MSIS definition of PJI in hip and knee arthroplasty.

Finally, this protocol helps establish bacterial colonization in upper extremity arthroplasty and, in this study, aid in identification of clinically significant colonization/infections that would have otherwise not been classified as infection before AIP implementation. Our AIP affected antibiotic administration and altered the microbiological diagnosis in 18 of 40 patients, yet the effect of clinical and surgical impression is still essential, as seen in the case of patient 39. Currently, no benchmark exists to define PJI, particularly shoulder arthroplasty. Historically, frozen section and operative cultures have improved the ability to detect infection,^29,30^ yet there remains a notable risk of error.^31^ There were no circumstances in our study where 3+ of the 5 cultures were not treated with antibiotics, and admittedly, it would be a very unlikely scenario where antibiotic treatment would be withheld in this situation. Barring some unusual circumstance, we would recommend against withholding antibiotics. Although a bit of a stretch, possibilities could include poor tolerance to antibiotic medication or a patient not willing to accept antibiotic treatment because of relatively minimal complaints with the prosthetic compared with perceived adverse effects of treatment. Conversely, a single patient who had negative cultures was still treated with antibiotics based on the history of previous infection and, therefore, fell outside of our protocol. Although we made every effort to adhere to the protocol, we recognize the unlikely necessity of clinical decision making overriding the scope of the AIP. Overall, we feel our current protocol generates another valuable point of reference in the diagnosis of suspected joint infections and provides new information useful for the treatment of these infections that would have otherwise been missed by older less comprehensive protocols.

The effect of the AIP on upper extremity arthroplasty revisions was larger than that in previous studies for lower extremity subsets (45% versus 30% change in antibiotic usage, respectively).^20^ Differences between the upper and lower extremities likely reflect the predominance of fastidious *Propionibacteria* as both a pathogen and contaminant in the upper extremity. Wee et al^11^ found an unexpected positive culture rate of 7.5% in a series of revision total elbow arthroplasties, with the majority being CoNS or *Propionibacteria*. Kelly et al^32^ found a 29% positive culture rate, primarily by *Propionibacteria*, at the time of presumed aseptic revision shoulder arthroplasty. Pottinger et al^16^ established a protocol for revision shoulder arthroplasty similar to our AIP, highlighting the importance of *Propionibacteria* and prolonged culture incubation. Although it can be argued that incubation for *Propionibacteria* could be as long as 14 days^23^ or even 28 days,^16^ the decision to hold prolonged cultures for 10 days was a compromise between hospital management and surgical staff. The balance between additional institutional cost and long-term savings for prudent use of antibiotics and lower reported hospital acquired infection rates remains undetermined and beyond the scope of this study. The AIP may not be fiscally prudent for patients who have a low likelihood of infection. A cost-benefit analysis stratifying patients by pretest probability for infection (history, examination, and infectious laboratory markers) is needed to better understand how to refine allocation of medical resources.

In summary, the AIP changed microbiological diagnosis and antibiotic treatment in 45% of revision shoulder and elbow arthroplasty cases and predicted joint sterility in 95% of culture-negative cases. For our institution, the protocol provided enough convincing benefit to warrant implementation as the standard of care for all shoulder and elbow revision arthroplasties.
